# Growth, Structure, and Photocatalytic Properties of Hierarchical V_2_O_5_–TiO_2_ Nanotube Arrays Obtained from the One-step Anodic Oxidation of Ti–V Alloys

**DOI:** 10.3390/molecules22040580

**Published:** 2017-04-05

**Authors:** María C. Nevárez-Martínez, Paweł Mazierski, Marek P. Kobylański, Grażyna Szczepańska, Grzegorz Trykowski, Anna Malankowska, Magda Kozak, Patricio J. Espinoza-Montero, Adriana Zaleska-Medynska

**Affiliations:** 1 Facultad de Ingeniería Química y Agroindustria, Escuela Politécnica Nacional, Ladrón de Guevara E11-253, P.O. Box 17-01-2759, Quito 170525, Ecuador; ma.cristina.nevarez@gmail.com; 2 Centro de Investigación y Control Ambiental “CICAM”, Departamento de Ingeniería Civil y Ambiental, Facultad de Ingeniería Civil y Ambiental, Escuela Politécnica Nacional, Ladrón de Guevara E11-253, P.O. Box 17-01-2759, Quito 170525, Ecuador; patricio.espinoza@epn.edu.ec; 3 Department of Environmental Technology, Faculty of Chemistry, University of Gdansk, Gdansk 80-308, Poland; marek.kobylanski@phdstud.ug.edu.pl (M.P.K.); anna.malankowska@ug.edu.pl (A.M.); magda.kozak@ug.edu.pl (M.K.); 4 Faculty of Chemistry, Nicolaus Copernicus University, Torun 87-100, Poland; gina@chem.umk.pl (G.S.); grazyna.szczepanska@umk.pl (G.T.)

**Keywords:** V_2_O_5_-TiO_2_ nanotubes, visible-light-driven photocatalysis, alloys, toluene degradation, air treatment

## Abstract

V_2_O_5_-TiO_2_ mixed oxide nanotube (NT) layers were successfully prepared via the one-step anodization of Ti-V alloys. The obtained samples were characterized by scanning electron microscopy (SEM), UV-Vis absorption, photoluminescence spectroscopy, energy-dispersive X-ray spectroscopy (EDX), X-ray diffraction (DRX), and micro-Raman spectroscopy. The effect of the applied voltage (30–50 V), vanadium content (5–15 wt %) in the alloy, and water content (2–10 vol %) in an ethylene glycol-based electrolyte was studied systematically to determine their influence on the morphology, and for the first-time, on the photocatalytic properties of these nanomaterials. The morphology of the samples varied from sponge-like to highly-organized nanotubular structures. The vanadium content in the alloy was found to have the highest influence on the morphology and the sample with the lowest vanadium content (5 wt %) exhibited the best auto-alignment and self-organization (length = 1 μm, diameter = 86 nm and wall thickness = 11 nm). Additionally, a probable growth mechanism of V_2_O_5_-TiO_2_ nanotubes (NTs) over the Ti-V alloys was presented. Toluene, in the gas phase, was effectively removed through photodegradation under visible light (LEDs, λ_max_ = 465 nm) in the presence of the modified TiO_2_ nanostructures. The highest degradation value was 35% after 60 min of irradiation. V_2_O_5_ species were ascribed as the main structures responsible for the generation of photoactive e^−^ and h^+^ under Vis light and a possible excitation mechanism was proposed.

## 1. Introduction

Over the past few decades, photocatalytic processes on the surface of TiO_2_ have been intensively studied due to a wide range of industrially oriented applications based on the conversion of sunlight into usable chemical energy [1,2,3,4,5,6]. Being non-toxic, abundant, chemically and physically stable, and photostable [7,8], TiO_2_ is a semiconductor material of great interest for environmental remediation [9,10], hydrogen evolution from water splitting [11,12], dye-sensitized solar cells [12,13], CO_2_ reduction [12,14], and self-cleaning surfaces [15,16]. However, the usage of TiO_2_ is limited not only by its wide bandgap (3.0–3.2 eV), which allows the absorption of only UV light corresponding to 4% of the incident solar energy [17], but also by the fast recombination rate of charge carriers [18,19]. In order to harvest sunlight, many TiO_2_ modification approaches have been developed [20,21], such as metal [22], nonmetal [23,24,25], or rare earth element doping [26], dye sensitization with organic and inorganic dyes [27], and the formation of photocatalytic heterostructures (coupling) with other semiconductors [28] or noble metals [29,30,31]. In particular, tuning TiO_2_ with V_2_O_5_ is an efficient way of improving TiO_2_ performance [32]. V_2_O_5_ is a small-bandgap semiconductor (~2.3 eV) which can extend the light absorption to the visible range [33]. Furthermore, photogenerated electrons and holes can be efficiently separated, and the surface charge carrier transfer rate is enhanced [34,35]. V_2_O_5_ itself has been used as a photocatalyst under UV light [36,37,38,39], visible light [40], and sunlight [41]. Xie, et al. [42] obtained photoactive V_2_O_5_-TiO_2_ nanocomposites for the oxidation of As(III). They stated that under visible light irradiation, h^+^ and O_2_^−^ are the main active species responsible for the photoreaction. Choi, et al. [43] synthesized V_2_O_5_-TiO_2_ nanocomposite powder by DC arc plasma. They found that, in the presence of the nanocomposite, Rhodamine B was decomposed under visible light, while it was not decomposed in the presence of TiO_2_ nanopowder. They also reported visible photoactivation and an enhanced charge separation in the case of toluene removal in a dielectric barrier discharge reactor. These aspects make the V_2_O_5_-TiO_2_ system an attractive material for visible-light-driven photocatalytic applications.

Moreover, TiO_2_ performance also critically depends on mass transfer, charge transfer, and charge/ion transport on its surface and bulk [7,44]. These processes are mainly controlled by morphology, which can be 0D (nanoparticles), 1D (nanowires, rods and tubes), 2D (layers and sheets), or 3D (spheres) [7]. Among 1D structures, TiO_2_ nanotubes (NTs) have become an interesting material because of their high electron mobility, excellent electron hole separation ability, long-distance transport capability, high specific surface area, mechanical strength, and extremely high aspect ratio [45,46]; however, no major improvement was reported for photocatalytic air purification with respect to nanoparticles under similar conditions [47].

Electrochemical anodization under specific conditions appears to be the simplest, least expensive, and most straightforward technique to obtain self-organized, auto-aligned NT arrays [48,49] over the surface of various metals, e.g., Ti [45,50,51], Zr [52], Hf [53], or alloys, e.g., TiNb, TiZr, TiTa [54], TiV [55,56], TiW [57], TiMn [58], TiMoNi [59], Ti_6_Al_4_V [60], and TiAg [61]. Anatase TiO_2_ nanotube array films with exposed {001} nanofacets, obtained by a low temperature hydrothermal method, exhibited enhanced UV activity, which was attributed to the enhanced charge separation derived from the synergy between {001} and {101} facets [62]. However, an electrochemical method is the most efficient for preparing mixed oxide nanotubes from a Ti suitable alloy. V_2_O_5_-TiO_2_ NTs have been successfully fabricated by electro-synthesis using Ti-V alloys as a substrate by the Schmuki research group [55] and Yang, Kim, Yang and Schmuki [56]. These mixed oxide NTs showed, respectively, improved electrochromic and capacitive properties compared with those of pure TiO_2_ NTs. Nevertheless, despite the proven visible light absorption of V_2_O_5_-TiO_2_ nanotubes, there is still a lack of data regarding the photoactivity of the V_2_O_5_-TiO_2_ NTs obtained from the anodization of Ti-V alloys. In our previous work [63], self-organized TiO_2_-MnO_2_ NTs were successfully obtained by the one-step anodization of Ti-Mn alloys in a fluoride-containing ethylene glycol (EG)-based electrolyte. The as-prepared layers were highly organized and showed visible-light photoactivity towards the degradation of toluene in the gas phase. It was demonstrated that a Vis-excited composite of wide and narrow bandgap oxides could be obtained by the anodization of Ti/V alloys, and that the preparation parameters (e.g., applied voltage, content of the MnO_2_ in nanocomposite) affected both the morphology and photoactivity of the TiO_2_/MnO_2_ NTs.

In view of this, this work focuses on the synthesis of visible-light photoactive V_2_O_5_-TiO_2_ NTs through the one-step anodic oxidation of Ti-V alloys in an ethylene glycol-based electrolyte, and their application in the photocatalytic degradation of toluene. The effect of the vanadium content in the alloy, applied voltage, and electrolyte composition (water content) was systematically studied to determine the influence of these parameters on the morphology and gas phase photoactivity, evaluated for the first time, of the obtained nanomaterials. The as-prepared NTs were characterized by using scanning electron microscopy (SEM), X-ray diffraction (XRD), energy-dispersive X-ray spectroscopy (EDX), micro-Raman spectroscopy, UV-Vis absorption, and photoluminescence spectroscopy. A possible mechanism of toluene degradation at the surface of V_2_O_5_-TiO_2_ NTs under the influence of visible light was also proposed.

## 2. Results and Discussion

### 2.1. Morphology and Growth Mechanism

**Figure 1:**
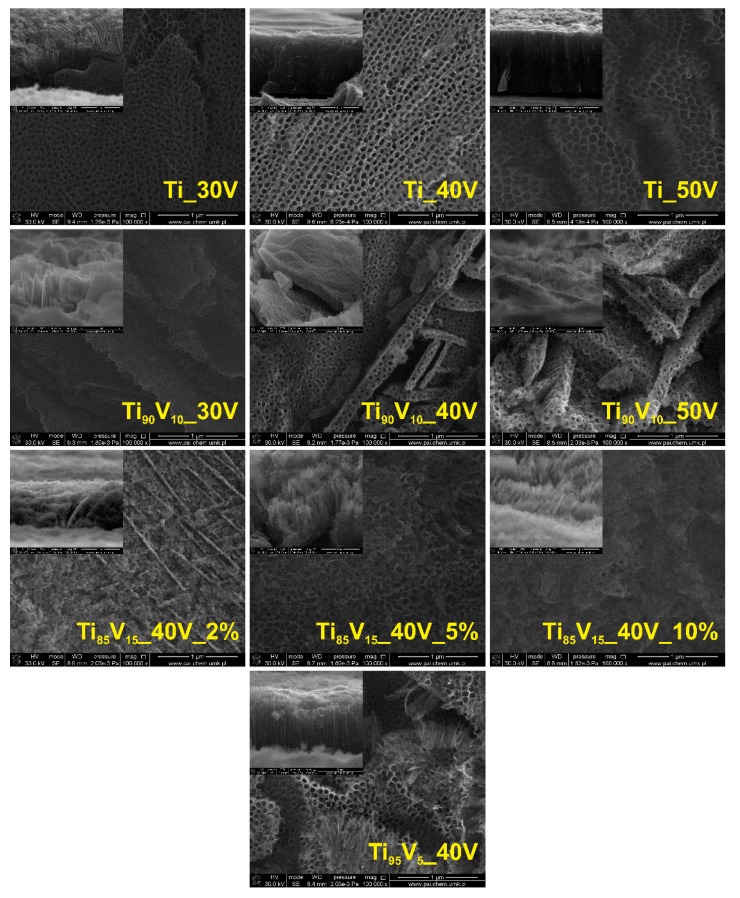
Top-view and cross-sectional scanning electron microscopy (SEM) images of pristine TiO_2_ nanotubes (NTs) and Ti-V anodized alloys.

Ti foils and Ti-V alloys of technical grade were anodically oxidized for 60 min, under the specific parameters summarized in Table 1. The effect of the applied potential (30, 40, and 50 V), vanadium content in the alloy (5, 10, and 15 wt %), and water content in the electrolyte (2, 5, and 10 vol %) on the morphology of the as-prepared samples were studied by scanning electron microscopy. The top-view and cross-sectional scanning electron microscopy (SEM) images are presented in Figure 1. The anodization of Ti sheets led to the formation of uniform and self-organized NTs with an open tube top and smooth walls, and the tube diameter and length ranged from 81 to 120 nm and from 1.5 to 16.2 μm, respectively (Ti_30V, Ti_50V, respectively). The samples anodized from the Ti-V alloys presented a different morphology, depending on the preparation parameters. The series of samples synthesized from alloys with a 10 wt % vanadium content generally exhibited a sponge-like structure integrated by overlapped layers with a tubular appearance. The registered diameters of these structures varied from 61 to 101 nm and the average thickness of the mixed oxide layers was 0.3–0.8 μm. The samples prepared from alloys with 15 wt % of vanadium and using electrolytes with different water contents showed different morphologies. The Ti_85_V_15__40V_2% and Ti_85_V_15__40V_10% samples presented a sponge-like structure made up of interconnected disordered bundles. Conversely, the Ti_85_V_15__40V_5% sample had a tubular structure with ripples on the tube wall, although the nanotubular layer was not highly organized. NTs presented a diameter (103 nm) similar to that of pristine TiO_2_ NTs (100 nm) obtained at the same voltage (40 V), while the length (0.9 μm) was smaller than that of the analogous pristine sample (5 μm). The highest level of self-organization was achieved with the sample obtained from the anodization of the alloy with a 5 wt % of vanadium content (Ti_95_V_5__40V), for which the synthesized NTs appeared to be composed of interconnected rings with a diameter of 86 nm and a length of 1 μm. As can be seen, the vanadium content in the alloy has a strong influence on the morphology of the samples. According to Yang, Kim, and Schmuki [55], the absence of a self-organized nanotube layer can be attributed to the low stability of the vanadium oxide, and therefore, the sample (Ti_95_V_5__40V) synthesized from the alloy with the lowest vanadium content exhibited the best auto-alignment and self-organization. The influence of the other parameters, applied potential and water content, on the morphology of the samples was not clear due to the strong influence of the vanadium content in the alloy.

**Figure 2:**
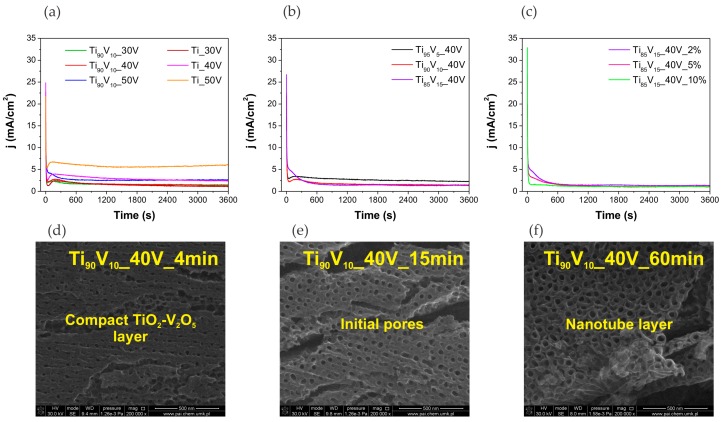
Current density-time curves recorded for the anodization of technical grade Ti foil and Ti-V alloys for the study of (**a**) applied voltage; (**b**) vanadium content in the alloy; and (**c**) water content in the electrolyte. SEM images of Ti_90_V_10__40V sample anodized during (**d**) 4 min; (**e**) 15 min; and (**f**) 60 min.

Considering these results, the SEM images of the Ti90V10_40V sample anodized during 4, 15, and 60 min (Figure 2d–f), together with literature data, a probable growth mechanism of V_2_O_5_-TiO_2_ NTs has been described. As can be seen in Figure 2a–c, the shape of the current density-time curves recorded for the V_2_O_5_-TiO_2_ samples were very similar to those of pristine TiO_2_ NTs. During the first stage, the formation of the V_2_O_5_-TiO_2_ oxide layer induced an exponential decrease in the current density, because of the reaction of Ti and V with the O_2_^−^ and OH^−^ ions from the water. The presence of this mixed oxide layer can be observed in Figure 2d, corresponding to the Ti90V10_40V sample after 4 min of anodization. Then, the current density progressively increased throughout the second stage due to the dissolution of the oxide layer, which led to an increase in the surface area of the electrode with the initiation of pore growth [64]. These soluble species correspond to the fluoride complexes, [TiF_6_]^2−^ and [VF_6_]^−^ [65,66]. Figure 2e shows the initial pores in the sample after 15 min of anodic oxidation. Finally, a regular and self-ordered NT layer, which can be appreciated in Figure 2f, is formed under a quasi-steady state, which is stablished due to the equilibrium between the formation and dissolution of the oxide layer. During this stage, pores equally share the available current [45].

**Figure 3:**
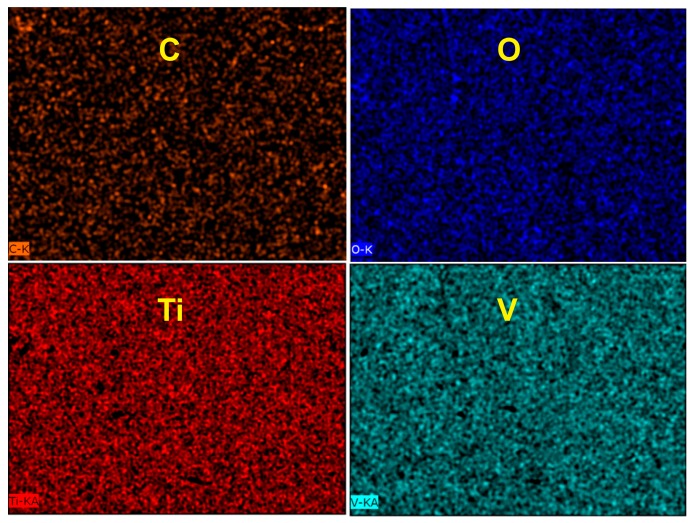
Energy-dispersive X-ray spectroscopy (EDX) mapping of the Ti_85_V_15__40V_5% sample.

The elemental composition of the obtained samples was analyzed through energy-dispersive X-ray spectroscopy (EDX) and the results presented in Table 1 show that the mass ratios between Ti and V in the V_2_O_5_-TiO_2_ mixed oxides nanostructures (NS) agree well with the nominal content of the alloy. In addition, no trace of elements other than Ti, V, C, and O, was observed. These findings confirm the chemical homogeneity of the nanotube layer. Furthermore, from the EDX mapping presented in Figure 3, it can be concluded that the aggregation of Ti and V was not observed.

### 2.2. Optical Properties

**Figure 4:**
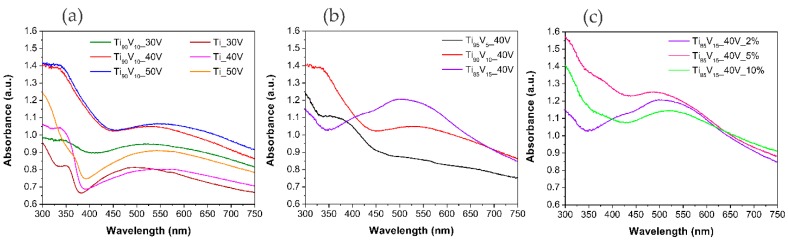
UV-Vis spectra of pristine TiO_2_ NTs and V_2_O_5_-TiO_2_ nanostructures (NS). Effect of (**a**) anodization potential; (**b**) vanadium content in the alloy; and (**c**) water content in the electrolyte.

The UV-Vis spectra of the obtained samples were compared with those of pristine TiO_2_ NTs. Figure 4a clearly shows that the samples prepared from Ti_90_V_10_ alloys exhibited a stronger absorbance in the broad visible range of 400–750 nm than TiO_2_ NTs. The spectra of the series with different vanadium contents, displayed in Figure 4b, indicated that an increase in the vanadium content in the alloy led to an increase in the absorbance intensity in the visible range, together with a red-shift. In particular, the spectrum of the sample Ti_85_V_15__40V presented a peak of maximum absorbance near 500 nm, which, according to literature data, corresponds to V_2_O_5_ [42,67]. The spectra of the series of samples prepared in an electrolyte with different water contents and plotted in Figure 4c are consistent with the previous statements and no clear effect of the water content on the UV-Vis properties was found. All of the spectra for this series of samples showed a peak of absorption in the Vis range near 500 nm, and the spectrum of the Ti_85_V_15__40V_5% sample showed the highest absorbance intensity peak. It can be concluded that the presence of the V_2_O_5_ in V_2_O_5_-TiO_2_ matrix enhanced the light absorption in the range of 400–750 nm.

**Figure 5:**
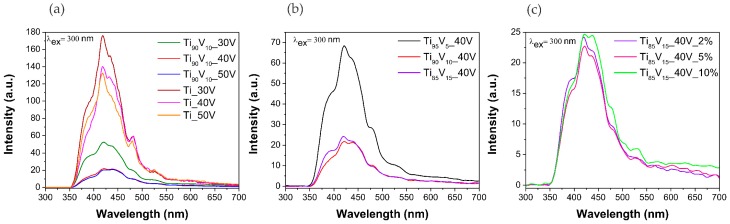
Photoluminescence spectra of pristine TiO_2_ NTs and V_2_O_5_-TiO_2_ NS. Effect of (**a**) anodization potential; (**b**) vanadium content in the alloy; and (**c**) water content in the electrolyte.

It is known that photoluminescence (PL) spectroscopy is a powerful tool for determining the presence of surface defects, trap states, and sub-band states in the mid-gap level of photocatalysts [68]. The PL spectra of the obtained photocatalysts are presented in Figure 5. It should be noted that the same emission and position peaks were observed among all series. Notably, the emission peak at approximately at 420 nm can be ascribed to the existence of self-trapped excitons from the TiO_6_^8−^ octahedron, while the two emission peaks at 450 and 485 nm could be assigned to the presence of surface defects, in the form of oxygen vacancies, which can create intermediate energy states located below the conduction band and which are able to trap electrons. The last peak at approximately 525 nm can be associated with the radiative recombination of the charge carriers [69,70].

The results mentioned above confirm the presence of surface/structural defects, which can play a role in the photocatalytic degradation of pollutants.

### 2.3. Structural Properties

**Figure 6:**
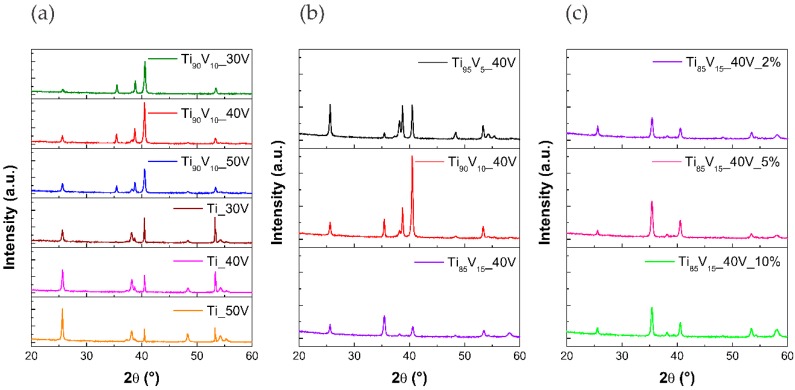
X-ray diffraction (XRD) spectra of pristine TiO_2_ NTs and V_2_O_5_-TiO_2_ NS. Effect of (**a**) anodization potential; (**b**) vanadium content in the alloy; and (**c**) water content in the electrolyte.

XRD patters of the obtained photocatalysts are presented in Figure 6. The calculated average crystallite size for pristine and modified TiO_2_ NTs are gathered in Table 1. The average crystallite size was calculated using the Scherrer equation, based on the (101) diffraction peak. In the registered region, peaks at 2θ values of 25.67°, 37.97°, 48.31°, 54.16°, and 55.30° can be ascribed to (101), (004), (200), (105), and (211) planes, respectively, which are characteristic of the anatase phase (JCPDS card). The other peaks at 2θ = 35.4°, 38.70°, 40.77°, and 53.31° can be ascribed to planes of metallic Ti substrate. As was mentioned above, the diffraction peaks corresponding to the pure anatase TiO_2_ phase were found, but other phases assigned to V_2_O_5_ were not observed. There are three possible explanations for this. Firstly, it could be because V_2_O_5_ diffraction peaks exist; however, the intensity of peaks is too low for this to be true. The absence of peaks corresponding to V_2_O_5_ in the XRD patters may be due to the low content and amorphous character of V_2_O_5_ or the short-range crystalline. Eventually, the vanadium species are incorporated into the TiO_2_ lattice. On the other hand, in modified samples, the bands assigned to the anatase phase had a smaller and wider intensity. In particular, the intensity of the pick ascribed to the characteristic (101) plane of anatase decreased with the increase in the vanadium content in the alloy. This is related to the smaller crystallite size of V_2_O_5_-TiO_2_ NS than that of pristine TiO_2_ NTs [71].

Furthermore, it can be seen that the intensity of the anatase reflexes increased, while those of the substrate decreased, with the increase of the anodizing voltage. This is caused by the increasing thickness of the nanotube layer.

The average crystallite size varied from 30 to 36 nm among Ti-V series, and from 33 to 38 nm for pristine TiO_2_ NTs. The smallest crystallite size was found for the Ti_90_V_10__50V sample, which reached 30 nm. A clear correlation between the crystallite size and (i) anodization potential; (ii) vanadium content in the alloy; and (iii) water content in the electrolyte, was not observed.

**Figure 7:**
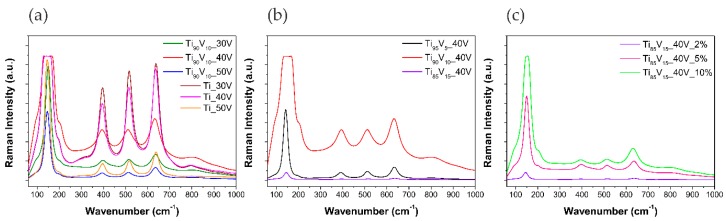
Raman spectra of pristine TiO_2_ NTs and V_2_O_5_-TiO_2_ NS. Effect of (**a**) anodization potential; (**b**) vanadium content in the alloy; and (**c**) water content in the electrolyte.

Micro-Raman spectroscopy was performed to determine the microstructure of the prepared samples. A 532 nm laser was used for the excitation. Figure 7 shows the Raman spectra of pristine TiO_2_ and V_2_O_5_-TiO_2_ NTs. The observed peaks at approximately 150, 396, 515, and 636 cm^−1^ are ascribed to the E_g_, B_1g_, A_1g_ + B_1g_, and E_g_ modes of the anatase phase, respectively, in agreement with previous reports [42,72,73,74]. The E_g_ modes are assigned to TiO_2_ symmetry, B_1g_ to O-Ti-O bending, and A_1g_ + B_1g_ to Ti-O stretching [75]. All of the spectra also registered a weak combination band at ca. 800 cm^−1^, which is characteristic of the Raman signature of anatase [76]. No distinguishable crystalline V_2_O_5_ Raman bands were present at 703 and 997 cm^−1^ in any spectra, probably due to the low content of vanadium in the alloy precursors or to the highly dispersed state of V_2_O_5_ in V_2_O_5_-TiO_2_ NS. This was also reported by former publications for composites with the V_2_O_5_-TiO_2_ system [32,75,77].

### 2.4. Photocatalytic Performance

**Figure 8:**
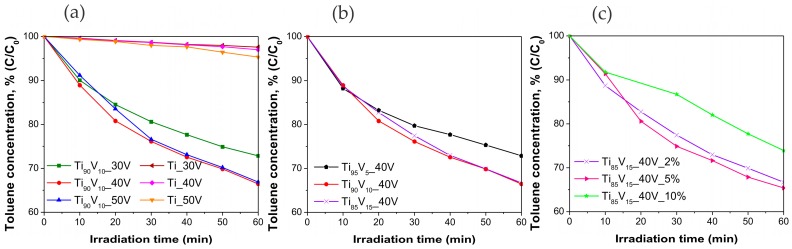
Photoactivity of pristine TiO_2_ NTs and V_2_O_5_-TiO_2_ NS in gas phase degradation of toluene under Vis-light irradiation (λ_max_ = 465 nm). Effect of (**a**) applied voltage; (**b**) vanadium content in the alloy; and (**c**) water content in the electrolyte.

The effect of the anodization voltage, vanadium content in the alloy, and water content in the electrolyte on the photocatalytic activity was evaluated through the degradation of toluene from an air mixture (200 ppmv of toluene) under Vis irradiation (LEDs array, λ_max_ = 465 nm). Figure 8 presents the degradation curves for the above-mentioned series and their comparison with the photoactivity of reference pristine TiO_2_ NTs. These plots show that V_2_O_5_-TiO_2_ samples from all series were active in the photodegradation reaction, in contrast with pristine TiO_2_ NTs which exhibited negligible toluene removal (ca. 5%). The highest degradation of toluene in the presence of samples prepared from the Ti_90_V_10_ alloys (see Figure 8a), after 60 min of irradiation, was observed for the sample anodized under 40 V (34%). The toluene removal reached by samples anodized under 30 V and 50 V were not that different from the best one (27% and 33%, respectively). In view of this, 40 V was selected as the potential for further synthesis, to determine the vanadium content in the alloy and the composition of the electrolyte solution, which are favorable for the photodegradation reaction. Figure 8b presents similar results, for the samples obtained from alloys with different vanadium contents. It can be observed that the vanadium content in the alloy slightly affected the photoactivity of the samples. The maximum toluene removal was found to be achieved for the sample with 10 wt % of vanadium in the alloy (Ti_90_V_10__40V, 34% of degradation). The analysis of the effect of water content in the electrolyte was carried out with NS obtained from Ti_85_V_15_ alloys. As can be seen in Figure 8c, there is a slight difference in the photocatalytic performance between these samples, among this series. The highest degradation of toluene was exhibited by the sample anodized in the electrolyte containing 5% of water and it corresponded to 35% of toluene removal (Ti_85_V_15__40V_5%). For a more detailed comparison of the obtained results, the initial reaction rate and reaction rate constants were calculated and presented in Table 1. The highest value for the initial reaction rate, among all series, was achieved in the presence of the Ti_85_V_15__40V_5% sample (7.08 × 10^−2^ μmol·dm^−3^·min^−1^), which also exhibited the highest absorbance intensity peak near 500 nm and consisted of a NT layer which was not highly organized. This suggests that this NT composite effectively enhanced visible light harvesting and the consequent photocatalytic reaction, owing to the presence of V_2_O_5_ [35,43]. Furthermore, no correlation between the morphology and the photocatalytic performance of the samples was observed.

In conclusion, the highest photoactivity under visible light (465 nm) was observed in the presence of the Ti_85_V_15__40V_5% sample. This sample not only exhibited the highest absorbance intensity at a wavelength of about 500 nm, but also reported the highest diameter (103 nm), the second longest NTs (0.9 μm), and the largest crystallite size (36 nm), from the modified samples. Its vanadium content, based on EDX analysis, was 9.08 wt %. On the other hand, the Ti_85_V_15__40V_10% sample showed the lowest photoactivity. It had a sponge-like morphology with a vanadium content of 8.91%, based on EDX analysis, which is lower than the content of the sample with the highest photoactivity, considering that both were prepared from Ti_85_V_15_ alloys. Its crystallite size was 32 nm, smaller than that of the Ti_85_V_15__40V_5% sample. The initial reaction rate achieved in the presence of this sample was 4.50 × 10^−2^ μmol·dm^−3^·min^−1^, which is 1.6 times lower than that reported for the most photoactive one (7.08 × 10^−2^ μmol·dm^−3^·min^−1^).

**Figure 9:**
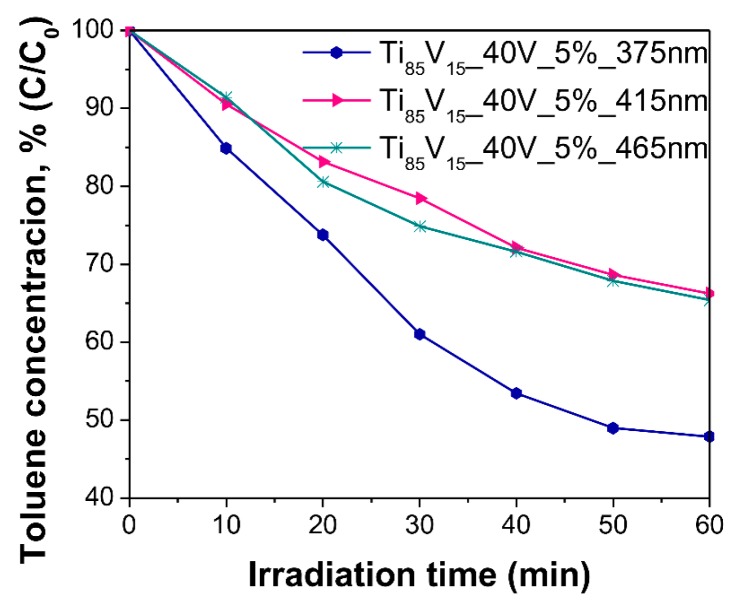
Photoactivity of Ti_85_V_15__40V_5% sample in gas phase degradation of toluene under different wavelengths of irradiation (λ_max_ = 375, 415, 465 nm).

**Figure 10:**
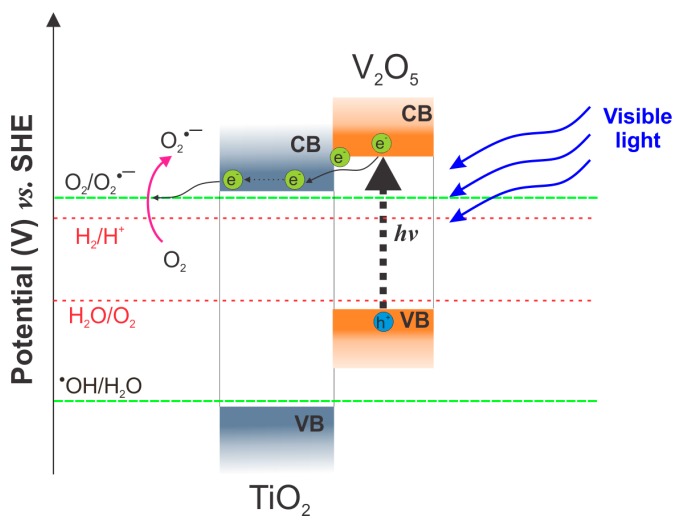
Excitation mechanism of V_2_O_5_-TiO_2_ samples under visible light irradiation.

To further analyze the photocatalytic properties of the synthesized composites, the effect of different irradiation wavelengths was studied using the most photoactive sample (Ti_85_V_15__40V_5%). The gas phase degradation of toluene was tested under 375, 415, and 465 nm and the obtained results are displayed in Figure 9. It can be observed that the highest degradation (52%) after 60 min of irradiation was achieved under UV light (375 nm). This can be explained by the presence of TiO_2_ in the NT matrix, which is the main active species under UV light irradiation. On the other hand, under the influence of visible light irradiation, 415 and 465 nm, the photocatalytic degradation reached almost the same level, in both cases, with values of 34% and 35%, respectively. This indicates that under Vis light irradiation, V_2_O_5_ are the main species responsible for the generation of e^−^ and h^+^ (as presented in Figure 10, excitation mechanism) over the surface of NTs, which led to the photodegradation of toluene, and this is supported by the negligible degradation reported for pristine TiO_2_ NTs under Vis light.

## 3. Materials and Methods

### 3.1. Materials

Acetone, isopropanol, and methanol were purchased from P.P.H. “STANLAB” Sp. J. (Lublin, Poland), ethylene glycol (EG) was acquired from CHEMPUR (Piekary Śląskie, Poland), and ammonium fluoride was bought from ACROS ORGANICS (Geel, Belgium). Technical grade Ti foils and Ti-V alloys with 5, 10, and 15 wt % of vanadium content were provided by HMW-Hauner Metallische Werkstoffe (Röttenbach, Germany). Deionized (DI) water with a conductivity of 0.05 μS was used to prepare all of the aqueous solutions.

### 3.2. Synthesis of Pristine TiO_2_ and V_2_O_5_-TiO_2_ Nanotubes

Ti foils and Ti-V alloys were ultrasonically cleaned in acetone, isopropanol, methanol, and deionized water for 10 min. Then, the foils were dried in an air stream. The anodization processes were carried out at room temperature, in an electrochemical cell consisting of a platinum mesh as the counter electrode, and the Ti-V alloy (2.5 cm × 2.5 cm) as the working electrode. A reference electrode of Ag/AgCl connected to a digital multimeter (BRYMEN BM857a, New Taipei City, Taiwan) was used to control and record information about the actual potential and current on the alloy. The anodization was conducted in an electrolyte composed of EG, water, and NH_4_F 0.09 M, during 60 min, with a voltage in the range of 30–50 V which was applied with a programmable DC power supply (MANSON SDP 2603, Hong Kong, China). Three electrolyte solutions with different water contents were used (volume ratios of EG:water of 98:2, 95:5, and 90:10). The obtained samples were rinsed with deionized water, sonicated in deionized water (1 min), dried in air (80 °C for 24 h), and calcined (450 °C, heating rate 2 °C/min) for 1 h.

### 3.3. Characterization of Pristine TiO_2_ and V_2_O_5_-TiO_2_ Nanotubes

The morphology of the synthesized pristine TiO_2_ and V_2_O_5_-TiO_2_ nanotubes was determined by using scanning electron microscopy (SEM, FEI QUANTA 3D FEG, FEI Company, Brno, Czech Republic). Energy-dispersive X-ray spectroscopy (EDX) analysis was performed with a scanning electron microscope (SEM, Zeiss, Leo 1430 VP, Carl Zeiss, Oberkochen, Germany). The crystal structure of the samples was determined from X-ray diffraction patterns recorded in the range of 2θ = 20°–90°, using an X-ray diffractometer (X’Pert Pro, Panalytical, Almelo, The Netherlands) with Cu Kα radiation. The crystallite size was calculated based on the Scherrer formula. Raman spectra were measured with a micro-Raman spectrometer (Senterra, Bruker Optik, Billerica, MA, USA) with a 532 nm excitation laser.

The UV-Vis absorbance spectra were registered on a SHIMADZU (UV-2600) UV-VIS Spectrophotometer (SHIMADZU, Kioto, Japan) equipped with an integrating sphere. The measurements were carried out in the wavelength range of 300–800 nm, the baseline was determined with barium sulfate as the reference, and the scanning speed was 250 nm/min at room temperature. The photoluminescence (PL) spectra were recorded at room temperature with a LS-50B Luminescence Spectrometer equipped with a Xenon discharge lamp as an excitation source and a R928 photomultiplier (HAMAMATSU, Hamamatsu, Japan) as detector. The excitation radiation (300 nm) was directed onto the surface of the samples at an angle of 90°.

### 3.4. Measurement of Photocatalytic Activity

The photocatalytic activity of the as-prepared NTs was analyzed, for the first time, in the purification of air from toluene, which was used as a model pollutant. The photodegradation experiments were carried out in a stainless-steel reactor with a volume of ca. 35 cm^3^. The reactor included a quartz window, two valves, and a septum. The light source consisting of an array of 25 LEDs (λ_max_ = 375, 415 and 465 nm, Optel, Opole, Poland) was located above the sample. The anodized foil was placed at the bottom side of the reactor and it was closed with the quartz window. A gas mixture (200 ppmv) was passed through the reactor during 1 min, the valves were then closed, and the reactor was kept in the dark for 30 min in order to achieve the equilibrium. Before starting the irradiation, a reference toluene sample was taken. The concentration was determined by using a gas chromatograph (TRACE 1300, Thermo Scientific, Waltham, MA, USA), equipped with an ionization flame detector (FID) and an Elite-5 capillary column. The samples (200 μL) were dosed with a gas-tight syringe for 10 min.

## 4. Conclusions

In summary, V_2_O_5_-TiO_2_ mixed oxide layers were successfully synthesized through the one-step anodization of Ti-V alloys in a fluoride-containing EG-based electrolyte. The obtained layers exhibited a sponge-like and nanotubular structure with highly enhanced optical and visible-light-photocatalytic properties, in contrast with pristine TiO_2_ NTs. The photoactivity of these anodically-obtained composites was evaluated for the first time in the degradation of toluene (200 ppmv) in the gas phase under visible light, with a twenty-five-LED array as the irradiation source (λ_max_ = 465 nm). All of the V_2_O_5_-TiO_2_ samples were reported as photoactive and the initial degradation reaction rate was in the range of 4.50–7.08 × 10^−2^ μmol·dm^−3^·min^−1^. The visible light harvesting was attributed to the presence of the narrow-bandgap V_2_O_5_ species in the matrix of the V_2_O_5_-TiO_2_ composites. A morphological study was also reported and the vanadium content in the alloy was found as the key factor limiting the self-ordering of the electrochemically prepared thin layers. The highest photoactivity under visible light (465 nm) was observed in the presence of the Ti_85_V_15__40V_5% sample. This sample not only exhibited the highest absorbance intensity at about 500 nm, but also reported the highest diameter (103 nm), the optimum length (0.9 μm), and the largest crystallite size (36 nm) among all of the modified samples. EDX analysis revealed that the vanadium content in this sample was equal to 9.08 wt %. In sum, the photocatalytic properties of these highly efficient nanocomposites, obtained through the most suitable method (electrochemical technique), permit new insights into the exploitation of industrially oriented applications, for instance, photocatalytic devices for air purification. The presented materials are photoactive under a low powered light source, and thus, the use of low cost light-emitting diodes (LEDs) as an irradiation source can significantly reduce the cost of photocatalytic air treatment processes, which is consistent with the principles of green chemistry.

## Figures and Tables

**Table 1:** Sample labels, preparation parameters, characterization, and photocatalytic activity of V_2_O_5_-TiO_2_ nanotubes under Vis irradiation.

Sample Label	Preparation Parameters		External Diameter (nm)	Tube Length (μm)	Wall Thickness (nm)	Average Crystallite Size (nm)	EDX Analysis				Photoactivity Vis Light (λ_max_ = 465 nm)	
	Electrolyte	Applied Potential (V)					Ti (wt %)	V (wt %)	C (wt %)	O (wt %)	Initial Reaction Rate × 10^2^ (μmol·dm^−3^·min^−1^)	Reaction Rate Constant × 10^3^ (min^−1^)
Ti_90_V_10__30V	EG 98% (*v*/*v*), H_2_O 2% (*v*/*v*), NH_4_F 0.09 M	30	61	0.8	13	31	74.61	7.61	0.01	17.78	5.34	5.98
Ti_90_V_10__40V	EG 98% (*v*/*v*), H_2_O 2% (*v*/*v*), NH_4_F 0.09 M	40	91	0.3	19	32	73.84	7.39	0.01	18.78	6.76	7.57
Ti_90_V_10__50V	EG 98% (*v*/*v*), H_2_O 2% (*v*/*v*), NH_4_F 0.09 M	50	101	0.4	30	30	69.34	6.85	0.01	23.79	6.56	7.35
Ti_85_V_15__40V_2%	EG 98% (*v*/*v*), H_2_O 2% (*v*/*v*), NH_4_F 0.09 M	40	Sponge-like structure			31	73.09	12.14	0.01	14.78	6.62	7.41
Ti_85_V_15__40V_5%	EG 95% (*v*/*v*), H_2_O 5% (*v*/*v*), NH_4_F 0.09 M	40	103	0.9	20	36	67.06	9.08	1.10	22.77	7.08	7.92
Ti_85_V_15__40V_10%	EG 90% (*v*/*v*), H_2_O 10% (*v*/*v*), NH_4_F 0.09 M	40	Sponge-like structure			32	66.10	8.91	1.14	23.86	4.50	5.04
Ti_95_V_5__40V	EG 98% (*v*/*v*), H_2_O 2% (*v*/*v*), NH_4_F 0.09 M	40	86	1.0	11	33	72.59	3.25	0.02	24.14	5.39	6.04
Ti_30V	EG 98% (*v*/*v*), H_2_O 2% (*v*/*v*), NH_4_F 0.09 M	30	81	1.5	10	33	71.47	0.00	0.19	28.34	0.37	0.42
Ti_40V	EG 98% (*v*/*v*), H_2_O 2% (*v*/*v*), NH_4_F 0.09 M	40	100	5.0	13	34	66.73	0.00	0.03	33.24	0.43	0.49
Ti_50V	EG 98% (*v*/*v*), H_2_O 2% (*v*/*v*), NH_4_F 0.09 M	50	120	16.2	18	38	67.69	0.00	0.03	32.28	0.64	0.72
